# Patterns of Posttraumatic Stress Disorder Symptoms and Posttraumatic Growth in an Epidemiological Sample of Chinese Earthquake Survivors: A Latent Profile Analysis

**DOI:** 10.3389/fpsyg.2018.01549

**Published:** 2018-08-28

**Authors:** Chengqi Cao, Li Wang, Jianhui Wu, Gen Li, Ruojiao Fang, Xing Cao, Ping Liu, Shu Luo, Brian J. Hall, Jon D. Elhai

**Affiliations:** ^1^Shenzhen Key Laboratory of Affective and Social Cognitive Science, Shenzhen University, Shenzhen, China; ^2^Key Laboratory of Mental Health, Institute of Psychology (CAS), Beijing, China; ^3^Department of Psychology, University of Chinese Academy of Sciences, Beijing, China; ^4^People's Hospital of Deyang City, Deyang, China; ^5^Global and Community Mental Health Research Group, University of Macau, Macau, Macau; ^6^Department of Health, Behavior and Society, Johns Hopkins Bloomberg School of Public Health, Baltimore, MD, United States; ^7^Department of Psychology, University of Toledo, Toledo, OH, United States

**Keywords:** posttraumatic stress disorder, posttraumatic growth, latent profile analysis, social support, natural disaster, DSM-5

## Abstract

**Background:** Posttraumatic stress disorder (PTSD) and posttraumatic growth (PTG) are different psychological outcomes triggered by exposure to extraordinarily traumatic events. In this study, we aimed to examine patterns of co-occurrence between DSM-5 PTSD symptoms and PTG, among natural disaster survivors, and to clarify whether empirically-based subgroups differed by several specific predictors.

**Methods:** Latent profile analysis was used to examine patterns of self-reported PTSD symptoms and PTG in an epidemiological sample of 1063 Chinese earthquake survivors.

**Results:** Three distinct profiles were identified, involving high PTSD/high PTG, mild PTSD/mild PTG, and mild PTSD/high PTG. Class membership was predicted by several variables, especially different sources of perceived social support.

**Conclusion:** Our findings contribute to the knowledge about the coexisting patterns of PTSD and PTG, and provide suggestions for identifying high-risk individuals and providing effective interventions in clinical practice.

## Introduction

According to the latest global epidemiological data, over 70.0% of people have been exposed to at least one traumatic event, with 30.5% reporting exposure to four or more (Benjet et al., 2016). Traumatic events can result in negative psychological emotional outcomes, and PTSD is the most common trauma-related mental disorder (Koenen et al., 2017). However, positive psychological changes, such as posttraumatic growth (PTG), have also been reported. PTG are positive changes that result from experiencing trauma (Tedeschi and Calhoun, 1995), and has been increasingly researched (Shakespeare-Finch and Lurie-Beck, 2014) with the development of positive psychology. However, little is known about subgroups of clinical presentations of posttraumatic symptoms and posttraumatic growth.

Both PTSD and PTG are posttraumatic reactions, and the relationship between them has been extensively discussed. However, the statistical relationship between these two constructs has resulted in mixed findings. Some studies found positive associations between PTSD and PTG (e.g., Tiamiyu et al., 2016; Liu et al., 2017; Zalta et al., 2017), while other studies reported that PTSD was inversely associated with PTG (e.g., Hall et al., 2008; Ssenyonga et al., 2013) or failed to find robust associations (e.g., Klosky et al., 2014). Moreover, a meta-analysis yielded both a linear, and a stronger curvilinear, relationship (Shakespeare-Finch and Lurie-Beck, 2014). Tsai et al. (2015) further revealed an inverted U-shaped non-linear relationship. Specifically, people with moderate levels of PTSD showed the highest levels of PTG. These mixed findings call for further research to resolve them.

Most of the previous studies mainly relied on variable-centered methodologies to investigate the relationship between PTSD and PTG, which may overlook individual differences within heterogeneous populations. Latent class analysis (LCA; examining categorical outcome variables) or latent profile analysis (LPA; examining continuous outcome indicators of symptom severity), are person-centered methods to classify individuals into latent groups based on similar patterns of observed variables (McCutcheon, 1987). To date, only three person-centered studies were conducted to explore the coexisting patterns of PTSD and PTG. The first LCA were conducted 10 months after the 2011 Oslo bombing attack, in two independent samples of individuals, one physically proximate to a bomb attack, and the other not physically proximate to the attack. Three classes of posttraumatic reactions were found. Specifically, a high PTSD/high PTG and mild PTSD/high PTG group were found in both of the two samples, while a mild PTSD/mild PTG group only existed in the group not physically proximate to the attack (Birkeland et al., 2015). Second, Chen and Wu (2017a) and Zhou et al. (2018) conducted LPA studies 8 and 12 months after earthquake respectively, to identify profiles of PTSD and PTG among Chinese adolescent earthquake survivors. They found similar three class patterns, characterized by mild PTSD/mild PTG, high PTSD/high PTG, and mild PTSD/high PTG. In addition to LPA/LCA studies, a recent latent transition analysis examined the transitions of different PTSD and PTG classes across two time points, finding 3-class patterns that were similar to those from (Chen and Wu, 2017a,b).

In summary, there are only limited empirical studies on the latent profiles of PTSD and PTG. Moreover, the existing person-centered studies assessed PTSD symptoms based on *DSM-IV* criteria. The *DSM-5* was released in 2013 with important additions and revisions of the PTSD symptom criteria. The new set of *DSM-5* PTSD symptoms changed the diagnosis (Hoge et al., 2014) and structure of PTSD symptom criteria (e.g., Liu et al., 2014), which may impact the profiles of co-occurring PTSD and PTG. Furthermore, the 21-item Posttraumatic Growth Inventory (PTGI; Tedeschi and Calhoun, 1996) is the most widely used scale to measure of PTG. However, the 2-item spiritual change factor of the PTGI has been criticized for its breadth of content coverage (Tedeschi et al., 2017). In response, Tedeschi et al. (2017) developed a new version of the PTGI - the PTGI-X - with 4 new items, adding a more comprehensive spiritual change factor to improve the assessment of spiritual growth in PTG. The impact of the revision of PTGI on the coexisting patterns of PTSD and PTG remains unclear. Therefore, additional studies are clearly needed to further clarify the relationship between PTSD and PTG, using more recent diagnostic criteria and newer assessment instruments.

To fill this gap, we conducted an LPA study in an epidemiological sample of Chinese adult earthquake survivors. Our first aim was to identify patterns of *DSM-5* PTSD symptoms and PTG in this sample. The second aim of this study was to examine whether memberships with different symptom profiles could be predicted by several specific predictors. It is well documented that trauma severity, social supports and demographic characteristics are associated with PTSD (e.g., Brewin et al., 2000) and PTG (e.g., Meyerson et al., 2011). Therefore, we further assessed the predicative functions of earthquake-related exposure, social support and demographic characteristics on subgroups with different symptom profiles. Furthermore, social supports included potentially distinct sources (e.g., Procidano and Heller, 1983; Zimet et al., 1988) and previous studies showed that different sources of social support have different prediction functions on PTSD (e.g., Nguyen et al., 2016) and PTG (Hasson-Ohayon et al., 2016). Accordingly, the third aim of this study was to further clarify the role of different sources of social support in predicting memberships with different symptom profiles.

## Materials and methods

### Participants and procedures

On May 12, 2008, a devastating earthquake measuring 8.0 on the Richter scale hit the Hanwang Town in Mianzhu City, China. The earthquake destroyed almost all the buildings there, leaving more than 5,000 people dead. The sample was recruited from rebuilt communities in Hanwang town, nine and a half years after the earthquake. Participants were adult earthquake survivors (aged 16 or older) without mental retardation or any major psychosis (e.g., schizophrenia and organic mental disorders). Trained investigators (e.g., clinical psychologists, psychiatrists, psychotherapists, and psychology graduate students) provided a detailed introduction of the aim and significance of the study, and then administered self-reported questionnaires to consenting participants. This study was approved by the Institutional Review Board of the Institute of Psychology, Chinese Academy of Sciences.

Of the 1074 respondents, 9 participants were removed from the sample as missing data were greater than 20%, and 2 participants were removed due to missing basic demographic data. The final valid sample included 1,063 participants. In this sample, 346 (32.5%) were males and 717 (67.5%) were females. The majority of participants were married (86.1%), with an average age of 51.1 years (*SD* = 10.0). Regarding educational attainment, 737 (69.3%) had less than a completed high school education, and 326 (30.7%) completed high school or higher education. The vast majority of participants (99.5%) were self-reported as Chinese Han ethnicity.

### Measures

Trauma exposure was measured with a 10-item commonly used questionnaire (Table 1). Respondents were instructed to answer yes (1) or no (0) regarding if they experienced a variety of traumatic event-related characteristics during the earthquake. The total score of the questionnaire was used to reflect the level of trauma exposure from the earthquake.

**Table 1:** Features of trauma exposure during the earthquake of the sample.

**Trauma exposure during the earthquake**	***n***	**%**
Were you trapped under rubble? (Yes)	127	11.9
Were you injured? (Yes)	150	14.1
Were you disabled due to injuries? (Yes)	42	4.0
Did you participate in rescue efforts? (Yes)	451	42.4
Did you witness a death of someone? (Yes)	712	67.0
Did you see mutilated bodies? (Yes)	417	39.2
Did any family members die of the disaster? (Yes)	299	28.1
Were any family members injuries?	398	37.4
Did any friend or neighbor die of the disaster? (Yes)	837	78.7
Did you lose your livelihood due to the disaster? (Yes)	383	36.0

PTSD symptoms were measured with the PTSD Checklist for DSM-5 (PCL-5; Weathers et al., 2013). The PCL-5 is a 20-item self-report scale corresponding to the DSM-5 PTSD symptom criteria. The five-point Likert-scale (0 = not at all to 4 = extremely) is used to reflect the severity of PTSD symptoms specifically in relation to the earthquake during the past month. The Chinese version of the PCL-5 was translated and back-translated from English, and has demonstrated good reliability and validity in Chinese samples (e.g., Liu et al., 2014; Wang et al., 2017). Based on the seven-factor model of PTSD (Armour et al., 2015), scores on intrusion, avoidance, negative affect, anhedonia, externalizing behaviors, anxious arousal, and dysphoric arousal factors were calculated. Cronbach's α for the scale in the sample was 0.95 for the total scale, and 0.89, 0.86, 0.84, 0.83, 0.71, 0.78, and 0.78 for intrusion, avoidance, negative affect, anhedonia, externalizing behaviors, anxious arousal, and dysphoric arousal, respectively.

Posttraumatic growth was measured with the Posttraumatic Growth Inventory-expanded (PTGI-X) (Tedeschi et al., 2017). The PTGI-X is a 25-item self-report measure, adapted from the original PTGI with 4 newly developed items in the spiritual change domain. Respondents were instructed to rate each item on a 6-point Likert scale to indicate the degree of change that occurred after the earthquake ranging from 0 (no change) to 5 (very great degree of change). The Chinese version of the original PTGI has demonstrated sound psychometric properties (e.g., Cheng et al., 2015; Liu et al., 2015). The Chinese version of the PTGI-X was translated based on the original PTGI-X via a two-stage process of translation and back translation. Based on the five-factor model of the PTGI (Tedeschi et al., 2017), scores for appreciation of life, personal strength, new possibilities, relating to others and spiritual and existential change factors were calculated. Cronbach's α for the scale was 0.95 for the total scale, and 0.64, 0.83, 0.81, 0.87, and 0.82 for appreciation of life, personal strength, new possibilities, relating to others, and spiritual and existential change, respectively, in the current sample.

Social support was measured with the Multidimensional Scale of Perceived Social Support (MSPSS). The MSPSS is a 12-item self-reporte scale composed of three subscales (i.e., family, friends, and significant others) (Zimet et al., 1988). Each item is rated on the 7-point Likert-scale ranging from 1 (very strongly disagree) to 7 (very strongly agree) to reflect the level of perceived social support. The Chinese version of the MSPSS was translated and back-translated from English, and has sound psychometric qualities (e.g., Zhou K. et al., 2015). Cronbach's α for the scale in the sample was 0.95. Cronbach's αs for family, friends and significant others were 0.89, 0.88 and 0.86 respectively.

### Data analysis strategies

Statistical analyses were conducted by Mplus 7.0. In this sample, 85 (8.0%) were missing one PCL-5 item, 25 (2.4%) were missing two or three PCL-5 items; 69 (6.5%) were missing one PTGI-X item, 35 (3.3%) were missing two to five items; 64 (6.0%) were missing one MSPSS item, and 7 (0.7%) were missing two items. Missing values on the PCL-5, PTGI-X and MSPSS were estimated with maximum likelihood (ML) procedures. The 3-step LPA method was conducted using maximum likelihood estimation. As described by Asparouhov and Muthen (2013), the LPA model was conducted only based on the PTSD and PTG scale scores in the first step. For the purpose of maximizing the interpretability of different solutions and facilitating model convergence (Au et al., 2013), we used seven PTSD factor observed scale scores (i.e., intrusion, avoidance, negative affect, anhedonia, externalizing behaviors, anxious arousal, and dysphoric arousal) (Armour et al., 2015) and five PTG factor observed scale scores (i.e., appreciation of life, personal strength, new possibilities, relating to others and spiritual and existential change) (Tedeschi et al., 2017) as indicators in this step. All 12 variables were converted to standardized T-scores to facilitate interpretation as differences exist in the scale range between the PCL-5 and PTGI-X. Latent class membership was calculated based on the latent class posterior distribution. In the final step, age, gender, educational level, marital status, trauma exposure, and social support were treated as independent variables to predict latent class membership. Odds ratios (ORs) with 95% confidence intervals were calculated based on beta values and standard errors. The dominant advantage of this 3-step method is that its multinomial regression analysis in the third step is carried out in consideration to minimize misclassification.

Two- to five-class models were conducted and compared based on a set of fit indices: (1) Bayesian information criterion (BIC) values and Akaike information criterion (AIC) values, with lower values indicating better model fit; (2) Lo–Mendell–Rubin likelihood ratio test (LMR LRT), adjusted LMR LRT (ALMR LRT) and bootstrapped likelihood ratio test (BLRT), with significant *P*-values indicating that a model with k classes fits the data better than a the model with *k*-1 classes. Moreover, entropy values closer to 1, indicating better classification accuracy, were considered. Finally, the parsimony and interpretability of the model were also taken into account to determine the best model.

## Results

Mean scores for the PCL-5 and PTGI-X were 19.0 (*SD* = 15.4; range: 0–80) and 68.7 (*SD* = 25.3; range: 0–125), respectively. By counting the numbers of symptoms endorsed at equal to or higher than a value of 2, probable PTSD diagnosis was determined based on the *DSM-5* diagnostic algorithmic scoring rules with at least one intrusion symptom, one avoidance symptom, two negative alterations in cognitions and mood symptoms, and two arousal symptoms. Based on the criteria, 170 (16.0%) participants would be identified as probable PTSD cases. Features of trauma exposure during the earthquake of the sample are presented in Table 1. The mean score of earthquake exposure was 3.6 (*SD* = 2.1; range: 0–10). Mean scores for social support from family, friends and significant others were 20.6 (*SD* = 5.5; range: 4–28), 19.5 (*SD* = 5.5; range: 4–28), and 19.4 (*SD* = 5.4; range: 4–28), respectively.

Fit indices of the four LPA models are presented in Table 2. The 3-class model yielded the best fit to the data, with lower AIC and BIC values, and significant LMR LRT, ALMR LRT, and BLRT values. Although the 4-class solution showed lower AIC and BIC values than the 3-class solution, the 4-class solution's non-significant LMR LRT and ALMR LRT values indicated that adding a new class to the 3-class model would not significantly improve model fit. Moreover, the higher entropy value for the 3-class solution than the 4-class solution suggests better classification accuracy. Therefore, the 3-class model emerged as the best fitting model based on model fit and parsimony.

**Table 2:** Fit indices for latent profile analyses.

	**AIC**	**BIC**	**Entropy**	**LMR LRT**	**ALMR LRT**	**BLRT**
2-class	91311.273	91495.121	0.933	<0.001	<0.001	<0.001
3-class	89289.160	89537.603	0.914	0.0032	0.0034	<0.001
4-class	88066.189	88379.227	0.912	0.2240	0.2262	<0.001
5-class	87363.526	87741.159	0.896	0.7028	0.7031	<0.001

*BIC, Bayesian Information Criterion; AIC, Akaike Information Criterion; LMR LRT, Lo–Mendell–Rubin likelihood ratio test; ALMR LRT, Lo–Mendell–Rubin adjusted likelihood ratio test; BLRT, Bootstrap likelihood ratio test*.

**Figure 1:**
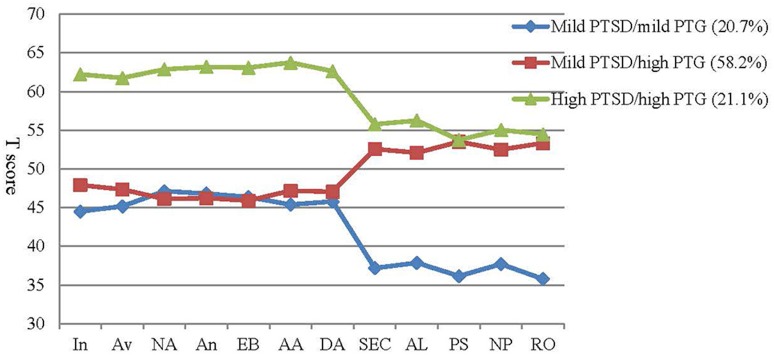
T-scores for subscales of the PCL-5 and PTGI-X for the 3-class model of symptom profiles. In, intrusion; Av, avoidance; NA, negative affect; An, anhedonia; EB, externalizing bahaviors; AA, anxious arousal; DA, dysphoric arousal; SEC, spiritual and existential change; AL, appreciation of life; PS, personal strength; NP, new possibilities; RO, relating to others.

The 3-class solution was characterized by mild PTSD/mild PTG (20.7%), mild PTSD/high PTG (58.2%) and high PTSD/high PTG (21.1%) patterns. T-score subscales for the PCL-5 and PTGI-X for the 3-class model's profile plot is presented in Figure 1. In the mild PTSD/mild PTG and high PTSD/high PTG groups, PTSD symptoms coexisted with comparable level of PTG. In the mild PTSD/high PTG group, individuals presented relatively low levels of PTSD symptoms and relatively high levels of PTG.

Results of the final step of the 3-step LPA for examining predictors of latent classes are shown in Table 3. Trauma exposure was a significant predictor of the three classes. Specifically, higher level of trauma exposure was associated with a higher probability of membership in mild PTSD/high PTG and high PTSD/high PTG group vs. mild PTSD/mild PTG group. Meanwhile, higher level of trauma exposure was also associated with a higher probability of membership in high PTSD/high PTG group than mild PTSD/high PTG group. Compared with the mild PTSD/high PTG group, individuals in the high PTSD/high PTG group were more likely to be older, female, less educated, and unmarried. Moreover, compared with the mild PTSD/mild PTG group, older people and females were more likely to be in the high PTSD/high PTG group. Regarding social support, more support from family was associated with a higher probability of membership in mild PTSD/high PTG versus high PTSD/high PTG group and mild PTSD/mild PTG group. Besides, more support from significant others was associated with higher probability of being classified in the mild PTSD/high PTG group than the mild PTSD/mild PTG group. Support from friends was not a robust predictor of class membership.

**Table 3:** Predictors of latent class groups.

	**Reference = mild PTSD/high PTG group**								**Reference = mild PTSD/mild PTG group**			
	**Mild PTSD/mild PTG group**				**High PTSD/high PTG group**				**High PTSD/high PTG group**			
	**B**	**SE**	**OR**	**95% CI**	**B**	**SE**	**OR**	**95% CI**	**B**	**SE**	**OR**	**95% CI**
Exposure severity	−0.182	0.056	0.83^**^	[0.72, 0.94]	0.258	0.050	1.29^***^	[1.20, 1.39]	0.439	0.070	1.55^***^	[1.41, 1.69]
FA	−0.126	0.030	0.88^***^	[0.82, 0.94]	−0.067	0.029	0.94^*^	[0.88, 0.99]	0.059	0.033	1.06	[1.00, 1.13]
FR	−0.044	0.029	0.96	[0.90, 1.01]	−0.014	0.028	0.99	[0.93, 1.04]	0.030	0.034	1.03	[0.96, 1.10]
SO	−0.062	0.028	0.94^*^	[0.89, 0.99]	0.001	0.031	1.00	[0.94, 1.06]	0.063	0.036	1.07	[0.99, 1.14]
Age	−0.008	0.011	0.99	[0.97, 1.01]	0.063	0.013	1.07^***^	[1.04, 1.09]	0.071	0.016	1.07^***^	[1.04, 1.10]
Sex	−0.215	0.208	0.81	[0.40, 1.21]	1.068	0.226	2.91^***^	[2.47, 3.35]	1.283	0.262	3.61^***^	[3.09, 4.12]
Education	−0.062	0.231	0.94	[0.49, 1.39]	−0.449	0.227	0.64^*^	[0.19, 1.08]	−0.388	0.277	0.68	[0.14, 1.22]
Marital status	0.245	0.284	1.28	[0.72, 1.83]	0.689	0.255	1.99^**^	[1.49, 2.49]	0.444	0.304	1.56	[0.96, 2.15]

*Sex was coded: 0, male; 1, female. Marital status was coded: 0, married; 1, divorced/separated/widowed/single. Education was coded: 0, less than high school; 1, high school and above. FA, supports from family; FR, supports from friends; SO, supports from significant others*.

* P < 0.05;

** P < 0.01;

*** *P < 0.001*.

## Discussion

The present study used LPA to identify patterns of *DSM-5* PTSD symptoms and PTG among Chinese adult earthquake survivors. 3-class pattern including mild PTSD/mild PTG, mild PTSD/high PTG and high PTSD/high PTG groups were identified, and were determined by demographic variables, trauma exposure and social support.

The 3-class profiles of this study were in accordance with existing LPA/LCA studies with a similar optimal fitting 3-class solution (e.g., Chen and Wu, 2017a; Zhou et al., 2018). As this study used the *DSM-5* criteria of PTSD, and the PTGI-X to measure PTG, the stable 3-class solution indicates that changes in the criteria of PTSD and revisions of the PTGI tend to not change the coexisting patterns of PTSD and PTG. It should also be noted that this study was conducted nine and a half years after the earthquake, which assessed the long-term psychological effects of trauma exposure. Previous LCA/LPA studies about this two constructs were carried out 8-12 months after disaster. Those similar results suggest that the coexisting patterns of PTSD and PTG are relatively stable across time.

A majority of individuals were in the mild PTSD/high PTG group in this study. This result was in line with a study of adolescent earthquake survivors showing that a considerable proportion of them obtained positive psychological changes (Chen and Wu, 2017a). According to a previous longitudinal study, PTG from previous traumatic events could buffer the adverse effects of subsequent traumas (Tsai et al., 2016). Therefore, these results highlight the potential for beneficial effects from, despite adverse exposure to traumatic events. Through the process of struggling with trauma, a majority of individuals not simply returned to the baseline, but experienced improvement beyond the previous status (Tedeschi and Calhoun, 2004). However, it should be noted that the perceived growth in this study measured by PTGI-X might reflect elements of illusory growth rather than actual growth (Boals and Schuler, 2018). When troubled by distress induced by traumatic event, individuals may report positive benefits of the experience by derogating their past self (McFarland and Alvaro, 2000). Experienced posttraumatic growth may reflect coping strategies used to manage distress. In line with this, perceived growth (but not actual) growth has been found to be related to positive reinterpretation (Frazier et al., 2009). Also, as the traumatic event happened nine and a half years ago, a retrospective bias may have influenced the reports of growth.

The results of this study also shed light on the controversial relationship between PTSD and PTG. Among the mild symptoms and high PTSD/high PTG groups, PTSD and PTG severity were reflective of each other. These findings indicate a positive association between PTSD and PTG, which are consistent with previous studies that have showed positive relationships (e.g., Tiamiyu et al., 2016; Liu et al., 2017; Zalta et al., 2017). In contrast, a negative association between the two constructs is shown in the mild PTSD/high PTG group, which is consistent with earlier research (e.g., Hall et al., 2008; Ssenyonga et al., 2013). These results indicate that population heterogeneity may be a confounding factor of mix findings.

An interesting phenomenon observed not only in this study but also in previous LPA/LCA studies was that the high PTSD/mild PTG group with high level of PTSD symptoms and low PTG levels was not identified. In other words, individuals with high level of PTSD symptoms always accompanied by relatively high PTG level. Two reasons may contribute to this result. On one hand, distressing emotions in individuals with PTSD symptoms can trigger a series of cognitive process to struggle with traumatic events, which will lead to PTG (Tedeschi and Calhoun, 2004; Meyerson et al., 2011). This idea was further supported by longitudinal studies showing that PTSD could predict follow-up PTG, but not vice versa (e.g., Zhou X. et al., 2015). On the other hand, the specific type of traumatic event may relate to this result. All of the LPA/LCA studies of PTSD and PTG were conducted in samples exposed to disasters (earthquakes and bombing attack). Studies have proved the role of traumatic event types in explaining PTSD symptoms (e.g., Kessler et al., 2017) and PTG (e.g., Gul and Karanci, 2017). Results of meta-analytic study (Shakespeare-Finch and Lurie-Beck, 2014) showed that the relationship between PTSD and PTG varied across traumatic event types, which implied that the patterns of PTSD and PTG may be different in various types of traumatic events. Further studies in other types of trauma (e.g., interpersonal violence, life-threatening illness) are needed to clarify whether there are other pattern groups of PTSD and PTG.

Determinants of symptom profiles were examined in this study. Regarding trauma exposure, higher level of trauma exposure was associated with higher probability of being classified into the pattern with a high level of PTSD (high PTSD/high PTG group) compared with low level of PTSD (mild PTSD/mild PTG group and mild PTSD/high PTG group). This results accord with studies showing that trauma severity could predict PTSD symptoms (e.g., Brewin et al., 2000). Meanwhile, higher level of trauma exposure was also associated with higher probability of being classified into the pattern with a high level of PTG (mild PTSD/high PTG group and high PTSD/high PTG group) compared with low level of PTG (mild PTSD/mild PTG group). This finding is in accordance with previous theory of PTG that a certain level of exposure is needed to trigger PTG (Tedeschi and Calhoun, 2004). The above observations reminder us that higher level of trauma exposure has both positive and negative effects on posttraumatic reactions.

The roles of demographic characteristics were also clarified. The results showed that females and older people were likely to enter the high PTSD/high PTG group. Females are known to engage in more ruminative thinking than males (Vishnevsky et al., 2010). Ruminative thinking may put people at greater risk for PTSD (e.g., Pineles et al., 2017). At the same time, ruminative thinking can also help people to be awareness of personal strengths or social connections to produce PTG (e.g., Tedeschi and Calhoun, 2004; Vishnevsky et al., 2010). Apart from ruminative thinking, females (e.g., Vingerhoets and Van Heck, 1990) and older people (e.g., Folkman et al., 1987) use more emotion-focused coping strategies. Emotion-focused coping strategies place one in high risk of PTSD (Lilly and Graham-Bermann, 2010). Meanwhile, PTG results from actively struggling to come to terms with the aftermath of the traumatic event (Tedeschi and Calhoun, 2004) and emotion-focused coping strategies embodies this process (Vishnevsky et al., 2010; Jin et al., 2014). Therefore, it is understandable that females and older people tended to have high PTSD symptoms and PTG. Less educated and unmarried was associated with higher probability of being classified into high PTSD/high PTG group compared with mild PTSD/high PTG group. These findings roughly accord with epidemiologic study showing that being unmarried and being less educated were associated with increased risk of PTSD (Koenen et al., 2017), Overall, these results provide implications for practitioners to identify individuals with different patterns of PTSD and PTG, and provide interventions for high risk individuals, especially those who are females, old, unmarried, and less educated.

The stress buffering theory dominates social support research (Lakey and Orehek, 2011) and the stress buffering effect of social support has been proved by many empirical researches (e.g., Hall et al., 2016; Zimmer-Gembeck and Skinner, 2016). Some studies have found that social support could predict the coexistence of PTSD and PTG (Wu et al., 2016) and the LPA study conducted by Chen and Wu (2017a) showed that higher level of social supports were more likely to be in the low PTSD/high PTG group compared with low PTSD/low PTG and high PTSD/high PTG groups. However, it should be mentioned that previous studies combined roles of multiple sources of support. This study was the first to examine different effects of various sources of support (i.e., family, friends, and significant others) on symptom profiles of PTSD and PTG. We found that family support was associated with membership in the mild PTSD/high PTG group compared with mild PTSD/mild PTG and high PTSD/high PTG group. It offers further support to the literature highlighting the preventive effect on PTSD (Soltani et al., 2015; Nguyen et al., 2016) and the facilitative role on PTG (Kimhi et al., 2009) of family support. The importance of family support may be due to the role of kinship (Nguyen et al., 2016) and the strength of familial ties within Chinese society (Lai, 2008). Apart from family support, support from significant others could differentiate individuals in the mild PTSD/high PTG group from those in the mild PTSD/mild PTG group while support from friends could not predict class memberships with different profiles. These results indicates that different sources of social support have distinct prediction effects on patterns of PTSD and PTG. The findings have implications for therapeutic practice by pointing the potential usefulness of improving support from family and significant others after natural disasters.

The theoretical and clinical implications of this study are noteworthy. First, combined with three other LPA/LCA studies of PTSD and PTG (Birkeland et al., 2015; Chen and Wu, 2017a; Zhou et al., 2018), this study improve our understanding different psychological reactions to trauma exposure. Additionally, the existence of different patterns of PTSD symptoms and PTG may help to explain the mixed findings of relationship between this two constructs. Specifically, in order to get a better understanding of posttraumatic psychological reactions, studies should take individual difference into consideration. This study also provide implications for clinical practice. Our results suggest that interventions targeting supports from family and significantly others would have potential benefits among natural disaster survivors. Moreover, this study provides implications to identify high risk individuals and give interventions individually tailored to their symptoms patterns.

Several limitations of this study should be mentioned. First, the findings of this study were based on self-report scales and need to be validated with further studies using clinical interviews. Second, the sample included Chinese adult earthquake survivors, which limits the generalizability of our results. Additional studies from different populations exposed to various trauma types are required to test the robustness of the pattern of profiles obtained in this study. Third, the cross-sectional design of this study precluded the examination of causality between PTSD and PTG. Additional prospective studies are needed to evaluate this relationship.

In spite of above-mentioned limitations, our study is the first to use a person centered approach to identify symptom patterns of co-occurring *DSM-5* PTSD and PTG in an epidemiological sample of Chinese adult earthquake survivors. The 3-class profiles of PTSD and PTG were revealed and determined by a number of variables, especially different sources of social support. Our findings add to limited literature on the coexisting patterns of these two constructs and may help to clarify mixed results from previous focusing on the association between PTSD and PTG. The present study also carry implications for clinical practice on which practitioners can identify high-risk individuals and provide effective interventions.

## Author contributions

CC conducted the literature review and the analysis, and wrote the first draft of the manuscript. LW conceptualized the study design, conducted the study, assisted with the analysis, and commented on drafts. JW, GL, RF, and XC assisted with the study and the analysis, and commented on drafts. PL and SL collected the data and commented on drafts. BH and JE commented on drafts. All authors have approved the final paper.

### Conflict of interest statement

The authors declare that the research was conducted in the absence of any commercial or financial relationships that could be construed as a potential conflict of interest.
